# Modulation of the Effects of Lung Immune Response on Bone Marrow by Oral Antigen Exposure

**DOI:** 10.1155/2013/474132

**Published:** 2013-09-22

**Authors:** P. Xavier-Elsas, C. L. C. A. Silva, L. Pinto, T. Queto, B. M. Vieira, M. G. Aranha, B. De Luca, D. Masid-de-Brito, R. A. Luz, R. S. Lopes, R. Ferreira, M. I. Gaspar-Elsas

**Affiliations:** ^1^Department of Immunology, Instituto de Microbiologia Paulo de Góes, Universidade Federal do Rio de Janeiro, Brazil; ^2^Department of Pediatrics, Instituto Nacional de Saúde da Mulher, da Criança e do Adolescente Fernandes Figueira (IFF/Fiocruz), Avenue Rui Barbosa, 716, Flamengo, 22250-020 Rio de Janeiro, CEP, Brazil

## Abstract

Allergic airway inflammation is attenuated by oral tolerization (oral exposure to allergen, followed by conventional sensitization and challenge with homologous antigen), which decreases airway allergen challenge-induced eosinophilic infiltration of the lungs and bone marrow eosinophilia. We examined its effects on bone marrow eosinophil and neutrophil production. Mice of wild type (BP-2, BALB/c, and C57BL/6) and mutant strains (lacking iNOS or CD95L) were given ovalbumin (OVA) or water (vehicle) orally and subsequently sensitized and challenged with OVA (OVA/OVA/OVA and H_2_O/OVA/OVA groups, resp.). Anti-OVA IgG and IgE, bone marrow eosinophil and neutrophil numbers, and eosinophil and neutrophil production ex vivo were evaluated. T lymphocytes from OVA/OVA/OVA or control H_2_O/OVA/OVA donors were transferred into naïve syngeneic recipients, which were subsequently sensitized/challenged with OVA. Alternatively, T lymphocytes were cocultured with bone marrow eosinophil precursors from histocompatible sensitized/challenged mice. OVA/OVA/OVA mice of the BP-2 and BALB/c strains showed, relative to H_2_O/OVA/OVA controls, significantly decreased bone marrow eosinophil counts and ex vivo eosinopoiesis/neutropoiesis. Full effectiveness in vivo required sequential oral/subcutaneous/intranasal exposures to the same allergen. Transfer of splenic T lymphocytes from OVA/OVA/OVA donors to naive recipients prevented bone marrow eosinophilia and eosinopoiesis in response to recipient sensitization/challenge and supressed eosinopoiesis upon coculture with syngeneic bone marrow precursors from sensitized/challenged donors.

## 1. Introduction

 The immunoregulatory effects of allergen exposure at the digestive tract, the major mucosal interface between the immune system and the antigens in environment, have received considerable attention over several decades [1, 2]. In the so-called *oral tolerization* models, feeding variable amounts of allergenic proteins to experimental animals, including mice, predictably changes their ability to subsequently respond to conventional sensitization and challenge with the same antigens, in a way consistent with attenuated, rather than exacerbated, hypersensitivity reactions [1–8]. Along with a variety of other strategies to change the course of allergic and autoimmune disease through immunomodulation, rather than through avoidance of environmental antigen exposure, oral tolerization holds promise for treatment as well as prophylaxis, and a better understanding of the mechanisms involved is likely to increase its practical value in management of immunological diseases [1, 2].

 One important, unexplored issue in this field is the mechanism through which oral tolerance affects eosinophilic inflammation, a hallmark of asthma as well as experimental models of allergic airway inflammation [9]. Eosinophils are recruited in large numbers to sites of allergen challenge in sensitized animals, and believed to participate in complex ways in the pathogenesis of asthma [9–11]. However, because they have a limited lifespan in tissues, which can be extended to some degree by a variety of inflammatory mediators [9], their relevance to the chronic manifestations of asthma depends on the ability of the bone marrow to continuously replace eosinophils that eventually undergo apoptosis, followed by degradation of apoptotic rests inside lung resident phagocytes [12]. Accordingly, the evidence from different models shows that an early consequence of airway challenge is upregulated eosinophil production in the bone marrow [13], paralleled by accumulation of eosinophil progenitors (colony-forming cells) in the lung tissue [14, 15]. 

While the impact of oral tolerization on bone marrow eosinophils has been examined by previous investigators, this effort has been limited, to our knowledge, to determining the percentage of eosinophils in bone marrow samples [3, 6]. This is, however, an unreliable indicator, because it can falsely increase or decrease following changes in the frequency of other bone marrow cell populations, regardless of any change in the numbers of eosinophils themselves. 

On the other hand, we have recently characterized two distinct mechanisms, operative in vivo, which effectively prevent the stimulatory effect of lung immune responses on bone marrow eosinophil production. The first requires both the inducible isoform of nitric oxide synthase (iNOS) and the cell surface-associated ligand for the so-called death receptor CD95 (Fas), CD95L [16]. The second involves suppression of eosinophil progenitors, paralleled by stimulation of neutrophil progenitors [17]. While the first mediates the effects of diethylcarbamazine in an experimental asthma model [16], the second is mobilized by G-CSF, a cytokine selectively stimulatory for neutrophils that has multiple immunoregulatory effects, including the ability to prevent upregulation of bone marrow eosinophil production by aerosol challenge of sensitized mice [17]. One, or both, of these mechanisms might be operative during oral tolerization and contribute to a reduction in eosinophil production, indirectly reducing eosinophilic inflammation. 

Given the complexity and interest of these interrelated issues, we have here examined whether (a) oral tolerization suppresses eosinophil production in the bone marrow, (b) it has additional effects on production of bone marrow neutrophils, or (c) a role for regulatory lymphocytes can be demonstrated.

## 2. Methods****

*Experimental Design and Groups*. Pilot studies established that tolerization only had a demonstrable effect on the bone marrow when oral exposure was followed by conventional sensitization and challenge. Accordingly, *oral tolerization* is hereafter defined as an initial oral exposure reinforced by conventional s.c. sensitization and by i.n. challenge, totaling 3 consecutive exposures in distinct anatomical sites (oral/subcutaneous/airway). In the *direct model* of oral tolerization, the full sequence of exposures (OVA/OVA/OVA) is both necessary and sufficient for characterization of hematological effects in vivo (see *Results*). Controls (nontolerized) are given only water in the first step (H_2_O/OVA/OVA) and thereafter are sensitized and challenged. Challenge controls receive saline (SAL) in the last step (OVA/OVA/SAL, H_2_O/OVA/SAL). In the *transfer (indirect) model*, the immunoregulatory mechanisms could be explored by inducing oral tolerization as above in a donor (OVA/OVA/OVA) mouse and by transferring this donor's splenic T lymphocytes into a naive syngeneic recipient, which subsequently underwent sensitization and challenge bypassing oral exposure. Recipient groups for the indirect model are termed T/OVA/OVA or OVA/T/OVA, indicating that T cell transfer occurred before sensitization or after sensitization, respectively. For OVA/T/OVA, transfer was done either *before* or *after* the boost injection.

*Mouse Strains and Animal Procedures*. Six- to 8-week-old mice of the BP-2, BALB/c (wild-type and CD95L-deficient, naturally occurring *gld* mutants, [16]), and C57BL/6 backgrounds (both wild-type and iNOS-deficient knockout mice, [16]), from CECAL-FIOCRUZ/RJ, were lodged and handled following institutionally approved (CEUA#L-010/04, CEUA#L-002/09) protocols. The original observations were made in BP-2; BALB/c and C57BL/6 mice were further used to examine the effect of different genetic backgrounds and of mutations in CD95L and iNOS. Where indicated, heparinized blood was collected for plasma antibody quantitation from the abdominal vena cava of mice anesthetized with an injection of xylazine (12 mg/kg) and ketamine (100 mg/kg), in a total 0.2 mL volume of saline.

*Immunological Reagents and Procedures*. For *oral tolerization*, mice were given dehydrated Hen Egg White (Salto's, São Paulo, Brazil) as a 1% (w/v) solution in drinking water for 5 consecutive days [3], changed once daily. Where indicated, bovine serum albumin or human serum albumin (BSA or HSA, Sigma-Aldrich, St. Louis, MO, both 1% w/v in drinking water) was given instead, to control for the antigen-specificity of oral tolerization. 

For *sensitization/boost*, mice were injected s.c. with 400 *μ*L saline containing 100 *μ*g Ovalbumin (OVA grade V, ICN Biomedicals, USA), the major antigen of Hen Egg White [18], and 1.6 mg alum, receiving two injections 7 days apart. For orally tolerized mice, sensitization and boost correspond to days 8 and 15, with oral exposure beginning at day 1. For* challenge*, they were given 25 *μ*L OVA 0.04% (w/v) in the nostrils at day 22. 

Anti-OVA IgG and IgE antibodies were quantified by ELISA as follows: flat bottom 96 well plates were sensitized overnight with 25 *μ*g OVA in 100 *μ*L PBS (1 h at 37°C followed by 4°C overnight). After 2 PBS washes, quenching was carried out for 1 h at 37°C with 5% Molico nonfat milk (for IgG) or BSA 1% (for IgE). Serum (100 *μ*L) diluted 1/800~1/25.600 (for IgG) or 1/500~1/16.000 (for IgE) in the respective quenching buffer was added for 1 h at 37°C, followed by 3 washes. Anti-murine IgG (Horseradish peroxidase-conjugated goat anti-mouse IgG, Southern Biotechnology Cat # 1031-05, 1/2000) or anti-murine IgE (rat anti-mouse IgE, Southern Biotechnology Cat # 1130-01, 1/1200, followed by biotinylated rabbit anti-rat IgG, Dako A/S, Denmark, Code # E0467, 1/1500, and avidin-HRP, eBioscience Lot E022539, 1/1500) antibodies were added for 1 h at 37°C for each step, with 3 PBS washes between incubations. The reaction was developed for 10 minutes with 100 *μ*L 0.1 M citrate-sodium phosphate buffer, pH 5.1, containing benzene-1,2-diamine (o-Phenylenediamine) 5 mg/10 mL and 4 *μ*L/10 mL 30% H_2_O_2_ (v/v) and stopped with 50 mL 4 N H_2_SO_4_. Absorbance was read at 490 nm. Where indicated, spleen cells were collected from tolerized and control donors, submitted to red cell lysis in Tris-NH_4_Cl for 2 minutes at 20°C, washed, and separated on Lymphoprep (density (20°C) 1.077 ± 0.001 g/mL, Nycomed Pharma AS, Oslo, Norway) cushions (300 ×*g*, 30 minutes, 20°C). Mononuclear cells from the interface were collected, washed, and filtered through nylon wool columns to yield T lymphocyte-enriched populations, recovered as 25–30% of the input. Where indicated, 10^7^ purified T cells were injected i.v. in a 100 *μ*L volume of PBS per recipient mouse through the tail vein. Sensitization of the recipients was started 24 h after transfer. Alternatively, T cells were used for coculture with hemopoietic precursors (see below) at a 1 : 10 ratio.

*Bone Marrow Assays*. Bone marrow was collected from both femurs of individual mice by flushing with RPMI-1640 medium containing 1% FCS. Total nucleated cell counts (in haemocytometer) and differential counts (on cytocentrifuge smears, stained for eosinophil peroxidase/EPO according to [19]) of EPO+ (eosinophil-lineage, encompassing both mature and immature) cells and neutrophils were carried out to determine the impact of oral tolerization on bone marrow in vivo. The ex vivo effects of oral tolerization were initially evaluated in *liquid *(nonclonal) bone marrow cultures, which allow the study of terminal eosinophil and neutrophil differentiation from committed precursors, established from 5 × 10^5^ freshly harvested bone marrow cells in 0.5 mL RPMI-1640 medium, with 10% FCS in the presence of IL-5 (eosinophil-selective) or GM-CSF (active on both the eosinophil and neutrophil lineages) and incubated at 37°C, 5% CO_2_/95% air, for 6 (GM-CSF) or 7 (IL-5) days, before counting total cells in a hemocytometer and determining the frequency of EPO+ cells and/or neutrophils in cytocentrifuge smears. These conditions were shown previously to support eosinophil proliferation and terminal differentiation and to allow detection of enhancing and suppressive effects [20, 21]. In the absence of exogenous IL-5 or GM-CSF, eosinophil differentiation does not occur, and cultures present virtually no EPO+ cells by day 7, containing only macrophages and endothelial/stromal cells, which survive from the bone marrow inoculum. 

Where indicated, bone marrow cells were separated (100 ×g, 20 minutes, 20°C) on Percoll (Sigma-Aldrich ref. St. Louis, MO) discontinuous gradients (40%/60%/75% isotonic Percoll in medium with 10% FCS). Cells in the 40%–60% interface (termed the P2 layer), enriched in hemopoietic precursors, including those of the eosinophil lineage, and depleted in mature eosinophils [13, 22] were washed and cultured as above, alone or together with T lymphocytes in a 10 : 1 ratio. 

 Further characterization involved *semisolid* (clonal) cultures, which allow definition of an impact on lineage-committed progenitors (colony-forming cells). These were established by seeding 2 × 10^5^ cells in 1 mL in 35 mm triplicate culture dishes in a mixture of IMDM with 20% FCS and agar Noble (0.3% final concentration) in the presence of GM-CSF (2 ng/mL). Colonies (defined as the progeny of a single progenitor, totaling at least 50 cells) were scored at day 7 under an inverted microscope [13, 20]. The frequency of eosinophil-containing colonies was determined on agar layers dried, mounted on microscope slides, stained for EPO, and scored under high magnification (400x). We have previously confirmed that these conditions were adequate for counting total myeloid colonies and for accurate differential counts of myeloid colony types on dried agar layers [13, 20].

*Statistical Procedures*. Comparisons between two groups were made by the two-tailed *t*-test; where indicated, multiple comparisons were made with ANOVA with the Tukey's (HSD) test or with Bonferroni's test. 

## 3. Results

**Figure 1:**
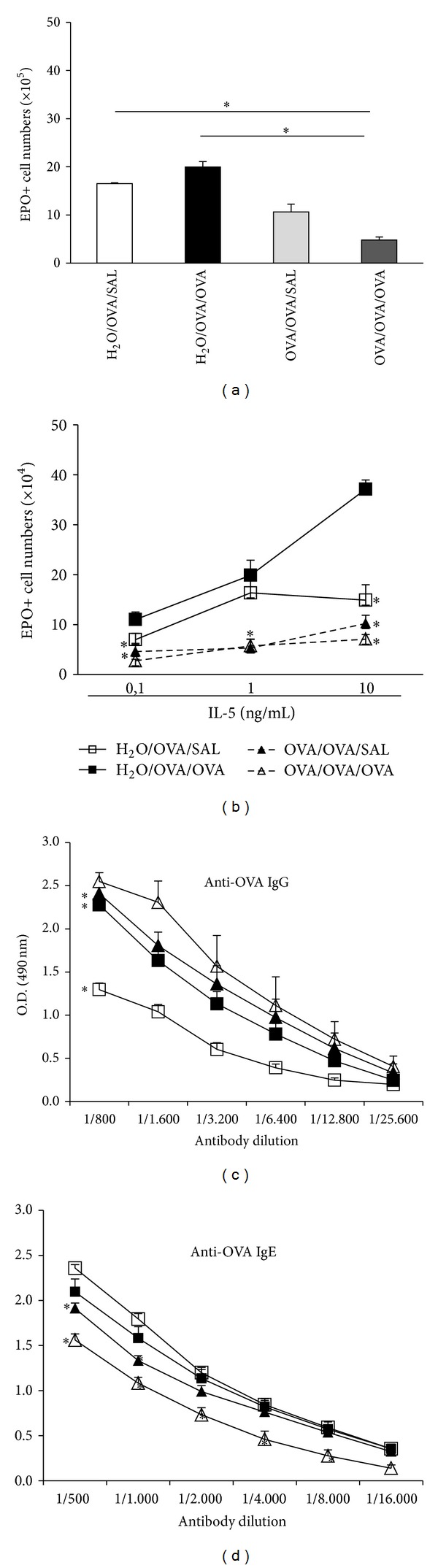
*Effects of oral tolerization on eosinopoiesis and anti-OVA antibody*. BP-2 mice, orally preexposed to OVA and further subcutaneously sensitized/boosted with OVA, were either saline challenged (OVA/OVA/SAL) or OVA challenged (OVA/OVA/OVA) by the intranasal route. Controls were given water, sensitized/boosted, and either saline challenged (H_2_O/OVA/SAL) or OVA challenged (H_2_O/OVA/OVA). Bone marrow ((a), (b)) and plasma ((c), (d)) were collected 24 h after-challenge. Data are Mean ± SEM. (a) EPO+ cell numbers in freshly collected BM (*n* = 6) (*P ≤ 0.004 for the indicated differences). (b) EPO+ cell numbers in bone marrow cultured with the indicated concentrations of IL-5 for 7 days. (*n* = 4) (*P ≤ 0.015 for the indicated differences relative to the respective control groups). ((c), (d)) Titration curves for IgG (c) and IgE (d) anti-OVA antibodies (*n* = 6) (*P < 0.01 for the differences relative to the respective control groups).

 We initially examined the effect of oral OVA preexposure on subsequent bone marrow and antibody responses to sensitization and challenge in mice of different strains. In BP-2 mice (Figure 1(a)), the numbers of EPO+ cells in freshly harvested femoral bone marrow, which reflect ongoing eosinopoiesis in vivo, were significantly different in tolerized-sensitized-challenged (OVA/OVA/OVA) mice relative to those of both sensitized (H_2_O/OVA/SAL) and sensitized-challenged (H_2_O/OVA/OVA) control groups. By contrast, tolerized-sensitized-unchallenged (OVA/OVA/SAL) controls, despite a trend towards lower eosinophil counts, did not significantly differ from either of the preceding control groups. Upon ex vivo exposure to IL-5 (Figure 1(b)), eosinopoiesis in bone marrow cultures from OVA/OVA/OVA mice was significantly different from the H_2_O/OVA/OVA controls at all concentrations of IL-5 tested. In this assay, response in OVA/OVA/SAL was significantly different from the respective H_2_O/OVA/SAL controls in the same IL-5 concentration range. In addition, challenge itself had a significant effect, at IL-5 10 ng/mL only (compare H_2_O/OVA/SAL and H_2_O/OVA/OVA). Hence, both in vivo and ex vivo assays demonstrate a significant effect of oral tolerization on subsequent responses of the bone marrow to S/C. Although the ex vivo assay may be more sensitive, full effectiveness in vivo seems to require sequential oral/subcutaneous/intranasal exposure. Oral tolerization did not prevent production of anti-OVA IgG (Figure 1(c)) or IgE (Figure 1(d)) antibodies. However, it shifted antibody class production towards increased IgG and decreased IgE production. As a consequence, the highest IgG/IgE ratios among all experimental groups were found in OVA/OVA/OVA mice. This coordinate shift in Ig class was considered evidence of immune deviation, rather than classical tolerance due to a failure of B lymphocyte activation.

**Figure 2:**
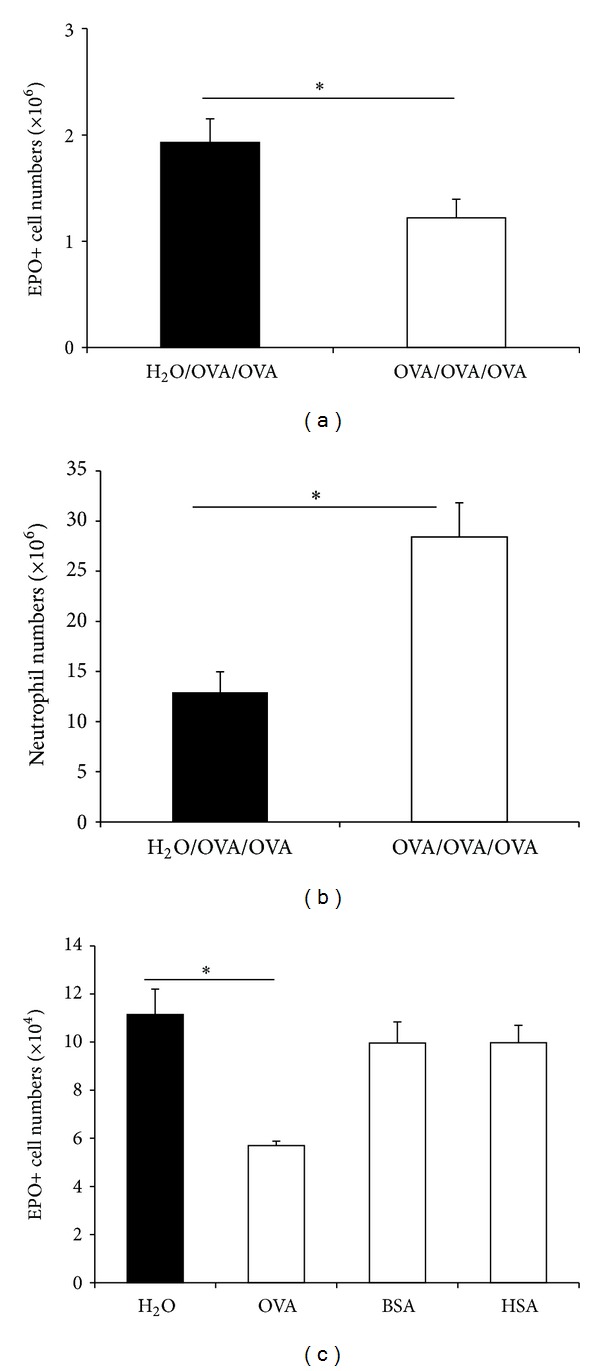
*Target- and stimulus-specificity of the effects of oral tolerization on BALB/c bone marrow*. BALB/c mice were orally preexposed to OVA ((a), (b)) or to the control antigens, BSA and HSA (c), before subcutaneous sensitization and airway challenge with OVA (open bars). Controls received only water before sensitization/challenge (black bars). Data (Mean ± SEM) are (a) EPO+ cell counts and (b) neutrophil counts, in freshly harvested bone marrow (*n* = 5) (*P ≤ 0.03); (c) EPO+ cell numbers in bone marrow cultured with 1 ng/mL IL-5 for 7 days (*n* = 3) (*P ≤ 0.001).

 We further examined the effects of this protocol in BALB/c mice, because a number of mutant strains of this background carry selective defects which can provide information on the mechanisms of bone marrow regulation by oral tolerance. Like BP-2 mice, BALB/c submitted to oral tolerization followed by sensitization and challenge (OVA/OVA/OVA) presented significantly reduced EPO+ cell counts in freshly harvested femoral bone marrow, compared with nontolerized H_2_O/OVA/OVA controls (Figure 2(a)). We also examined whether oral tolerization affected further the neutrophil lineage, which is closely related to eosinophils [23] but usually present in larger numbers in bone marrow and blood and stimulated by infection rather than by allergen exposure. In BALB/c mice, freshly harvested bone marrow from OVA/OVA/OVA donors presented significantly increased neutrophil counts relative to the H_2_O/OVA/OVA controls (Figure 2(b)). Furthermore, OVA/OVA/OVA BALB/c mice presented diminished ex vivo responses to IL-5 in bone marrow culture, as shown by significantly reduced EPO+ cell recovery relative to the same controls (Figure 2(c)). Importantly, tolerization to unrelated antigens (Figure 2(c); BSA and HSA, resp., third and fourth columns), followed by sensitization and challenge with OVA, failed to suppress eosinophil production in IL-5-stimulated cultures, highlighting the need for sequential oral/subcutaneous/respiratory exposure to the *same* antigen. Taken together, these observations pointed to a broad effect of oral tolerization on bone marrow, affecting neutrophils as well as eosinophils, and prompted further examination of granulopoiesis in vivo and ex vivo in the same strain originally used (BP-2).

**Figure 3:**
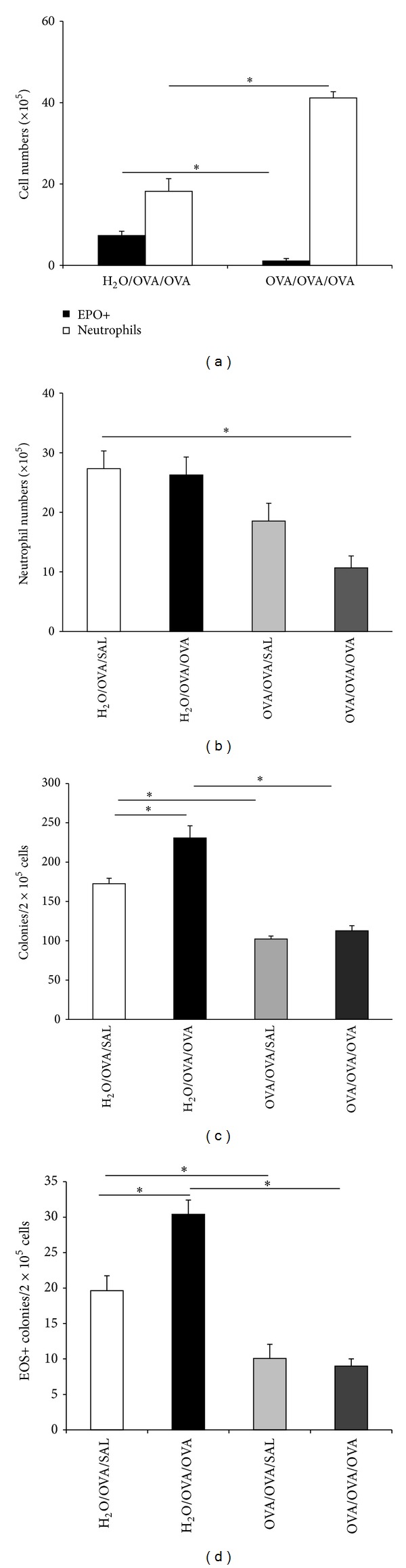
*Coordinate regulation of eosinophil and neutrophil numbers by oral tolerization.* Experimental groups of BP-2 mice were the same as in Figure 1. Data (Mean ± SEM) are (a) numbers of EPO+ cells (black bars) and neutrophils (white bars) in freshly harvested femoral bone marrow of OVA/OVA/OVA and H_2_O/OVA/OVA mice (*n* = 3); *P ≤ 0.003 for the indicated differences. (b) Numbers of neutrophils recovered after 6 days from liquid bone marrow cultures established with GM-CSF from the indicated groups *n* = 4; *P* = 0.005. (c) Total numbers of colonies formed by bone marrow from the indicated groups in the presence of GM-CSF (*n* = 6); *P ≤ 0.02 for the indicated differences. (d) Counts of colonies containing EPO+ cells from the experiment shown in (c), *n* = 6; *P* ≤ 0.009 for the indicated differences.

 In BP-2 mice, freshly harvested bone marrow from OVA/OVA/OVA donors presented significantly increased neutrophil counts relative to the H_2_O/OVA/OVA controls, along with significantly decreased EPO+ cell counts (Figure 3(a)). Hence, in BP-2 as well as in BALB/c mice (Figures 2(a) and 2(b)), bone marrow eosinophil and neutrophil counts were both affected in vivo by oral tolerization, although in opposite senses. We next examined whether these changes would be paralleled by changes observable ex vivo, when bone marrow was cultured with GM-CSF, a hemopoietic cytokine that, unlike IL-5 (see Figure 1(b)), supports differentiation of both eosinophils and neutrophils (Figures 3(b)–3(d)). Contrary to expectations, GM-CSF-stimulated neutrophil production in liquid culture was significantly reduced in cultures from OVA/OVA/OVA BP-2 mice, relative to the H_2_O/OVA/SAL and H_2_O/OVA/OVA nontolerized controls (Figure 3(b)). A less marked reduction, which did not reach statistical significance, was also observed in the OVA/OVA/SAL unchallenged controls, relative to the preceding nontolerized control groups. We further explored the effects of oral tolerization on GM-CSF-stimulated neutropoiesis, using colony formation assays to evaluate the responses of myeloid progenitors, which include both neutrophil (GM-, G-) and eosinophil (Eos-) colony forming cells (CFC), as well as mixed (GMEos [23]) CFC. Separate effects of oral tolerization could be detected in these conditions, depending on whether scores were of total myeloid colony numbers (Figure 3(c)) or of eosinophil-, neutrophil-, and macrophage-containing colonies (Figure 3(d)), determined by differential counts on stained agar layers. Myeloid colony formation of all types responsive to GM-CSF was significantly reduced in OVA/OVA/OVA bone marrow relative to the nontolerized control groups (Figure 3(c)). Myeloid colony formation was as effectively suppressed in OVA/OVA/SAL bone marrow as in mice submitted to the full sequence of allergen exposures (OVA/OVA/OVA). This clearly contrasts with observations of neutrophil production in liquid culture (Figure 3(b)) from the same animals, a discrepancy that may reflect the different sensitivity of the liquid versus semisolid culture conditions to the oral tolerization protocol. Importantly, differential counts showed the formation of eosinophil-containing (i.e., the sum of those formed by Eos- and GMEos-CFC) colonies to be significantly reduced, relative to the nontolerized control groups, in cultures from OVA/OVA/OVA as well as OVA/OVA/SAL BP-2 mice (Figure 3(d)). On the other hand, the formation of neutrophil-containing (i.e., the sum of G-, GM-, and GMEos-CFC) colonies was also reduced in the OVA/OVA/OVA and OVA/OVA/SAL groups, relative to nontolerized controls, mostly accounted for by halving of GM-CFC counts (not shown). Because these effects of oral tolerization on neutropoiesis and eosinopoiesis in semisolid culture do not require the full OVA/OVA/OVA sequence, they clearly contrast with the reduction of eosinophil counts in freshly harvested bone marrow, which was observed in this strain only in OVA/OVA/OVA mice (Figure 1(a)). 

 One mechanism for blocking the stimulatory effects of lung allergen challenge on eosinophil production, both in vivo and in bone marrow culture, requires iNOS and CD95L, acting sequentially [16]. Because such blockade resembles that achieved by oral tolerization in the present study, insofar as both prevent the impact of airway challenge on the bone marrow, we evaluated the relevance of iNOS and CD95L to the phenomena described above. In a separate set of control experiments (not shown), CD95L-deficient mice of the BALB/c background, submitted to the OVA/OVA/OVA protocol, presented downregulation of eosinophil production in vivo that was comparable to that observed in the respective wild-type controls in the same conditions (Figure 2(a)); total myeloid colony counts from semisolid clonal cultures were significantly reduced in OVA/OVA/OVA iNOS-deficient mice of the C57BL/6 background, relative to H_2_O/OVA/OVA controls of the same strain; suppression of eosinopoiesis in liquid culture from iNOS-deficient OVA/OVA/OVA mice, as compared to H_2_O/OVA/OVA controls, was also observed. Together, these argue against the involvement of iNOS or CD95L in the hemopoietic effects of oral tolerization. 

**Figure 4:**
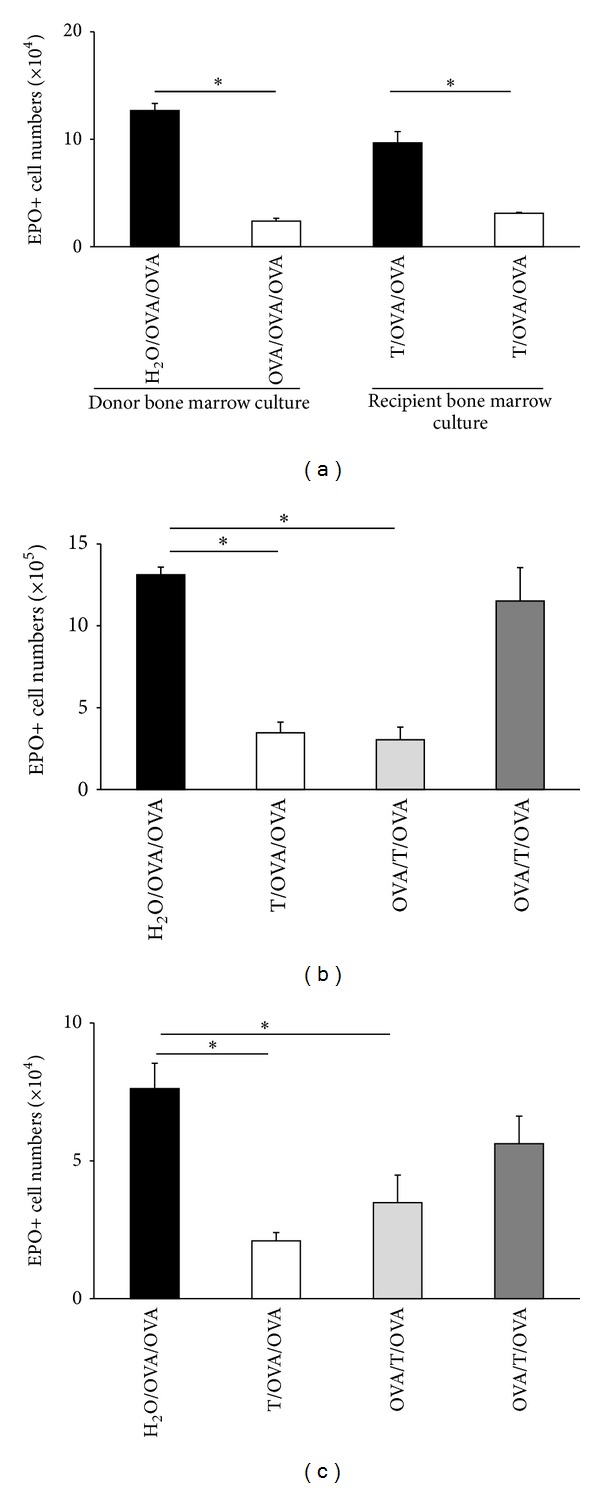
*Effect of splenic T lymphocytes on bone marrow eosinopoiesis in a transfer protocol*. T lymphocytes were isolated from spleens of BP-2 *donor* mice (either OVA/OVA/OVA tolerized or H_2_O/OVA/OVA controls) and used for i.v. transfers into syngeneic recipients. *Recipient *mice were either naïve and subsequently sensitized and challenged (T/OVA/OVA) or sensitized before transfer (OVA/T/OVA), challenge occurring after transfer. Data are Mean + SEM of EPO+ cell counts in 7-day IL-5 stimulated bone marrow cultures ((a), (c)) or freshly harvested bone marrow (b). (a) Eosinophil production ex vivo from donor (left) and recipient mice (right). T/OVA/OVA mice received T cells from control (black column) or tolerized (white column) donors. *n* = 6; *P* ≤ 0.025 for the indicated differences; (b), (c) EPO+ cell counts in bone marrow (b) and eosinophil production ex vivo (c) in control H_2_O/OVA/OVA mice (black columns) or in recipients of T cells from OVA/OVA/OVA donors, with transfer before sensitization (T/OVA/OVA, white bars), after sensitization but before boost (OVA/T/OVA, light grey bars), or after sensitization and boost (OVA/T/OVA, dark grey bars). *n* = 3; *P* ≤ 0.002 (b); *P* ≤ 0.036 (c) for the indicated differences.

 On the other hand, the increase in OVA-specific IgG/IgE ratios induced by oral tolerization, suggestive of class-specific regulation of antibody production by T lymphocyte subpopulations, prompted us to examine a possible contribution of T cells to the complex changes in bone marrow from OVA/OVA/OVA mice. This was examined in transfer experiments, in which T lymphocytes from OVA/OVA/OVA donors were injected i.v. into naïve syngeneic recipients, which were subsequently sensitized and challenged (Figure 4). This experimental design allowed us to separate the *induction* of oral tolerization from the *expression* of its immunoregulatory effects, because oral allergen exposure only occurred in the donor, while its effects mediated by T lymphocytes were detectable in the recipients, which were themselves never orally exposed to ovalbumin.

As shown in Figure 4, splenic T lymphocytes from OVA/OVA/OVA donors, upon transfer into syngeneic (BP-2) recipients, duplicated the effects of oral tolerization on the bone marrow. Confirming previous observations (Figure 1(b)), oral tolerization in the *direct model* significantly reduced eosinophil production ex vivo in IL-5-stimulated cultures established from bone marrow of tolerized and control donor mice (Figure 4(a), left). When spleen T cells from donors of these two groups were transferred into naïve recipients, which were themselves subsequently sensitized and challenged ((Figure 4(a), right), a significant reduction in eosinophil production ex vivo was seen in bone marrow from recipients of T cells from OVA/OVA/OVA donors, relative to H_2_O/OVA/OVA controls, showing that this effect of oral tolerization can be duplicated in mice that were never orally exposed to OVA, by merely providing the appropriate T cell population. Importantly, the same effect was observed in freshly harvested bone marrow (Figure 4(b)), confirming its relevance to hemopoiesis in vivo. As further shown (Figures 4(b) and 4(c)), T cell transfer from the appropriate donor retained its effectiveness in the recipient even after the sensitization injection (compare the light grey columns to the white columns in Figures 4(b) and 4(c)). By contrast, transfer after the boost injection (dark grey columns in Figures 4(b) and 4(c)) was no longer able to significantly decrease in vivo (Figure 4(b)) or ex vivo (Figure 4(c)) EPO+ cell counts relative to the respective H_2_O/OVA/OVA controls. 

**Figure 5:**
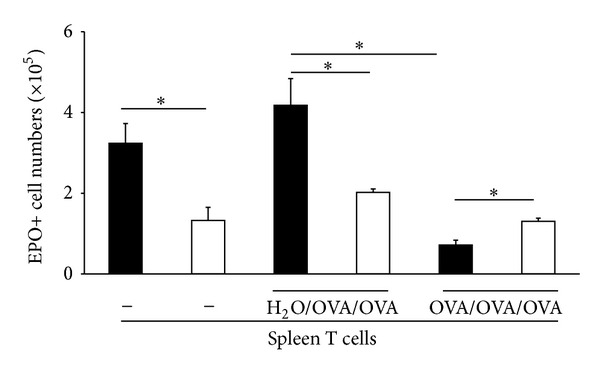
*Splenic T lymphocytes from tolerized donors suppress eosinopoiesis from bone marrow of sensitized/challenged mice in coculture*. Bone marrow and spleen cells were collected from OVA/OVA/OVA donors (white columns) and H_2_O/OVA/OVA (black columns) controls. Eosinophil precursors were separated on Percoll discontinuous gradients and cultured in the presence of IL-5 alone (−) or in coculture with splenic T lymphocytes from either experimental group as indicated, in a 10 : 1 ratio. Data are Mean + SEM of EPO+ cell numbers in 7-day cultures (*n* = 3, *P ≤ 0.02 for the indicated differences).

Finally, we examined whether splenic T lymphocytes would be able to influence eosinopoiesis ex vivo when co cultured with hemopoietic precursors. As shown in Figure 5, eosinophil production by hemopoietic precursors (fraction P2) isolated on Percoll discontinuous gradients [22] was significantly reduced in bone marrow from OVA/OVA/OVA donors relative to H_2_O/OVA/OVA controls. Addition of T lymphocytes from control H_2_O/OVA/OVA donors to the culture did not restore the eosinopoietic activity of precursors isolated from tolerized OVA/OVA/OVA donors. By contrast, addition of T lymphocytes from tolerized donors significantly reduced the eosinopoietic activity of precursors isolated from control H_2_O/OVA/OVA mice. Together, these observations suggest that oral tolerization induces long-term modification of eosinophil precursors, which may be directly evoked by coculture of nontolerized cells with T lymphocytes from tolerized donors.

## 4. Discussion

This is, to our knowledge, the first detailed description of the effects of oral tolerization on bone marrow hemopoietic function, both in vivo and ex vivo. It clearly establishes a parallelism between the previously reported modulation of lung immune responses by oral allergen preexposure [3] and a corresponding decrease in the production of eosinophils by the bone marrow. It further documents unexpected effects of oral tolerization on neutrophil numbers and neutrophil production. These effects suggest that shared immunoregulatory mechanisms coordinately regulate both the eosinophil and neutrophil lineages, even though the changes observed are not necessarily in the same direction in both lineages. 

We will address below a number of issues that warrant further investigation and the reasons thereof are as follows.

- *The role of oral, subcutaneous, and respiratory exposures to the same antigen*. In vivo, the eosinophil lineage was significantly suppressed only in animals which underwent three consecutive exposures to ovalbumin. This is important, because it shows that merely feeding allergen, without further sensitization and challenge, has no detectable impact on bone marrow composition. This is consistent with everyday experience in the consumption of food that contains variable amounts of antigenic proteins, which have no known impact on basic hematological parameters. Equally important is the observation that the antigen used for tolerization must be the same as that used for sensitization and challenge, in order to have a detectable effect in vivo. The need for identity of stimuli at the first and second step strongly suggests that the hematological effect is mediated by antigen-specific (clonal) cell populations and consistent with the evidence that this effect can be duplicated by T lymphocytes, both in transfer protocols and in coculture. The simplest interpretation is that the oral exposure primes a cell population that does not become active effector at this step, but becomes so by further encountering the same antigen at the sensitization and challenge steps. The implication is that such cell population does not stay restricted to the site of priming (presumably the gut-associated lymphoid tissue [1]) but rather circulates freely, as required for a restimulation with antigen injected subcutaneously in the dorsum. Evidence available so far suggests that it does not become, by this restimulation, able to suppress eosinophil production in vivo, but it may be already capable of interfering with the response of eosinophil precursors to IL-5 ex vivo. The implication would be that the effector cell, even before an airway challenge, is able to affect bone marrow cells. As a nonspecific, systemically active mechanism of action is unlikely, in view of the requirement for specific antigen, we suggest that after restimulation by conventional immunization, we assume that the effector cell does so in close proximity to its targets, which requires the ability to home to the bone marrow, where it can act on eosinophil precursors and progenitors, as shown by data on liquid and semisolid culture, respectively. This is consistent with evidence that T lymphocytes from the appropriate donors can, in coculture with the susceptible targets (precursors from nontolerized donors), effectively suppress eosinophil production. Overall, both the magnitude and apparent selectivity of the suppressive effects in vivo are consistent with the mechanism proposed to account for a decreased eosinophilia of the lung in tolerized animals [3]. They are equally consistent with observations on bone marrow eosinophilia in other models of oral tolerance involving peripheral allergen challenge at sites distinct from the lungs [6].
- *The nature and relevance of the effects on the neutrophil lineage*. Two kinds of effects were detected in the bone marrow neutrophils: (a) an in vivo increase in neutrophil numbers, consistent with descriptions of previous studies [6]; (b) an ex vivo decrease in neutrophil production, but in liquid and semisolid culture conditions. The latter could not have been detected in previous studies, which did not examine hemopoiesis, but rather focused on the percent eosinophils and neutrophils in bone marrow samples, which reflect not only production of these granulocyte types, but their rates of emigration from the bone marrow reserve pool as well as their longevity. Percent data can also be artifactually changed by increases or decreases in other bone marrow subpopulations. These pitfalls were avoided by the experimental approach we have taken, and we were able to clearly distinguish between an increase in absolute neutrophil numbers in vivo and a reduced production of neutrophils ex vivo in response to a standard cytokine (GM-CSF) stimulus. We favor the view that the in vivo data are more biologically relevant, and therefore can rule out that neutropenia and abnormal susceptibility to infection result from the oral tolerization protocol. We think, nevertheless, that the ex vivo data on reduced responses to GM-CSF are informative on important cellular events and might shed some light on the mechanisms of bone marrow regulation by oral tolerance.
- *The relationship of the effects on the eosinophil lineage to previously described mechanisms*. The observations in this study are inconsistent with the previously described mechanism involving sequential activation of iNOS and CD95L, since oral tolerance was very effective in mutant bone marrow lacking either iNOS or CD95L. By contrast, the effects of oral tolerance involve a decrease in eosinophil-containing colony formation, along with an increase in neutrophil numbers in vivo. This is consistent with observations in mice exposed to G-CSF in vivo, in which eosinophil production and eosinophilic inflammation were reduced, while the neutrophil lineage was stimulated [17]. A possible role for G-CSF in our experimental models, both direct and indirect, should be explored in future studies.
- *Ex vivo events can be linked to defective responses through βc-associated receptors*. One of the puzzling observations of this study is that neutrophils are increased in number in vivo, but neutrophil colony formation in the presence of GM-CSF ex vivo is reduced by oral tolerization. These apparently incompatible observations can be reconciled if one takes into account that GM-CSF is not required for neutrophil production in vivo, although it is a powerful stimulus for neutrophil colony formation in culture and most likely plays a role in the stress neutropoiesis associated with infection [24]. Therefore, both sets of observations can be reconciled if oral tolerization decreased the effectiveness of exogenous GM-CSF as a stimulus in culture, but not the production of neutrophils in vivo, which goes unabated even when bone marrow is insensitive to GM-CSF [24]. Interestingly, formation of eosinophil colonies in the presence of GM-CSF and eosinophil differentiation in the presence of IL-5 were both decreased. In this case, reduction of eosinopoiesis ex vivo was paralleled by decreased eosinophil numbers in bone marrow. Again, this requires understanding of the relationship of eosinopoiesis to both IL-5 and GM-CSF. These cytokines signal through distinct receptors, which share a common signaling *β* chain (*β*c; [24]). Cells in the eosinophil lineage express both IL-5 and GM-CSF receptors, signaling through which is abolished in *β*c-deficient mice. Although GM-CSF is not essential for neutrophil nor eosinophil production, IL-5 signaling is a major determinant of eosinophil production in vivo, and *β*c-deficient mice lack normal numbers of eosinophils [24]. All of our observations would therefore be consistent with a mechanism through which oral tolerization prevented expression or interfered with function of the *β*c chain. The predictions of this hypothesis would be the following: (a) eosinophils would be similarly reduced both in vivo and ex vivo, since *β*c-dependent signaling through IL-5 receptors is required in both settings; (b) *β*c expression would be reduced or absent from cells in both the eosinophil and neutrophil lineages, recovered from bone marrow of OVA/OVA/OVA mice but not of H_2_O/OVA/OVA controls.
- * The contribution of T lymphocytes to the regulation of bone marrow eosinophils*. In many studies of oral tolerance, an important role for T lymphocytes has been demonstrated [1, 2, 7, 8, 25–28]. In many cases, these have been isolated from gut-associated lymphoid tissue, including mesenteric lymph nodes, but regulation by oral tolerization-induced splenic T lymphocytes has also been demonstrated by others [26] as well as in the present study. There is considerable variability among different published studies as to the precise phenotype and properties of the immunoregulatory T cells involved [25–28], and at this point there is no obligate profile for a regulatory T cell in an oral tolerance model. As a consequence, the phenotype of a T cell responsible for suppressing eosinopoiesis and possibly increasing neutropoiesis must be established experimentally, rather than theoretically predicted on the basis of findings in other models and laboratories. The limiting factor, in this respect, is the availability of an assay that can accurately and reproducibly detect the relevant cells. While a T cell transfer protocol (Figure 4) is very useful and should be used for addressing questions that can only be answered in vivo (such as, for instance, whether or not they migrate to bone marrow, and which activating steps must occur before this happens), it is unfortunately limited, from a practical point of view, by the requirement for relatively large numbers of cells (10^7^ per recipient). We suggest that a coculture assay (Figure 5) circumvents these limitations, since it reproducibly detects activity with a much smaller number of T cells (5 × 10^4^) and allows parallel testing of many variables in a short time and with use of fewer animals. This is the assay that we are currently using to determine the phenotype and mechanism of action of the oral tolerization-induced T cells on bone marrow eosinopoiesis.

## References

[1] Weiner HL, da Cunha AP, Quintana F, Wu H (2011). Oral tolerance. *Immunological Reviews*.

[2] Faria AMC, Weiner HL (2006). Oral tolerance: therapeutic implications for autoimmune diseases. *Clinical and Developmental Immunology*.

[3] Russo M, Nahori M, Lefort J (2001). Suppression of asthma-like responses in different mouse strains by oral tolerance. *American Journal of Respiratory Cell and Molecular Biology*.

[4] Shin J, Kang JM, Won Kim S, Cho J, Park YJ, Kim SW (2010). Effect of oral tolerance in a mouse model of allergic rhinitis. *Otolaryngology*.

[5] Vaickus LJ, Bouchard J, Kim J, Natarajan S, Remick DG (2010). Oral tolerance inhibits pulmonary eosinophilia in a cockroach allergen induced model of asthma: a randomized laboratory study. *Respiratory Research*.

[6] Rodrigues CM, Martins-Filho OA, Vaz NM, Carvalho CR (2006). Systemic effects of oral tolerance on inflammation: mobilization of lymphocytes and bone marrow eosinopoiesis. *Immunology*.

[7] van Esch BCAM, Schouten B, de Kivit S (2011). Oral tolerance induction by partially hydrolyzed whey protein in mice is associated with enhanced numbers of Foxp3^+^ regulatory T-cells in the mesenteric lymph nodes. *Pediatric Allergy and Immunology*.

[8] Du Pré MF, Kozijn AE, Van Berkel LA (2011). Tolerance to ingested deamidated gliadin in mice is maintained by splenic, type 1 regulatory T cells. *Gastroenterology*.

[9] Rothenberg ME, Hogan SP (2006). The eosinophil. *Annual Review of Immunology*.

[10] Lee JJ, Dimina D, Macias MP (2004). Defining a link with asthma in mice congenitally deficient in eosinophils. *Science*.

[11] Humbles AA, Lloyd CM, McMillan SJ (2004). A critical role for eosinophils in allergic airways remodeling. *Science*.

[12] Cyr MM, Denburg JA (2001). Systemic aspects of allergic disease: the role of the bone marrow. *Current Opinion in Immunology*.

[13] Gaspar Elsas MIC, Joseph D, Elsas PX, Vargaftig BB (1997). Rapid increase in bone-marrow eosinophil production and responses to eosinopoietic interleukins triggered by intranasal allergen challenge. *American Journal of Respiratory Cell and Molecular Biology*.

[14] Gaspar Elsas MIC, Maximiano ES, Joseph D, Bonomo A, Vargaftig BB, Xavier Elsas P (2003). Isolation and characterization of hemopoietic cells from lungs of allergic mice. *Chest*.

[15] Xavier-Elsas P, Santos-Maximiano E, Queto T (2007). Ectopic lung transplantation induces the accumulation of eosinophil progenitors in the recipients’ lungs through an allergen- and interleukin-5-dependent mechanism. *Clinical and Experimental Allergy*.

[16] Queto T, Xavier-Elsas P, Gardel MA (2010). Inducible nitric oxide synthase/CD95L-dependent suppression of pulmonary and bone marrow eosinophilia by diethylcarbamazine. *American Journal of Respiratory and Critical Care Medicine*.

[17] Queto T, Vasconcelos ZFM, Luz RA (2011). G-CSF suppresses allergic pulmonary inflammation, downmodulating cytokine, chemokine and eosinophil production. *Life Sciences*.

[18] Mine Y (1995). Recent advances in the understanding of egg white protein functionality. *Trends in Food Science and Technology*.

[19] Horton MA, Larson KA, Lee JJ, Lee NA (1996). Cloning of the murine eosinophil peroxidase gene (mEPO): characterization of a conserved subgroup of mammalian hematopoietic peroxidases. *Journal of Leukocyte Biology*.

[20] Gaspar Elsas MIC, Maximiano ES, Joseph D (2000). Upregulation by glucocorticoids of responses to eosinopoietic cytokines in bone-marrow from normal and allergic mice. *British Journal of Pharmacology*.

[21] Xavier Elsas P, Neto HAP, Cheraim AB (2004). Induction of bone-marrow eosinophilia in mice submitted to surgery is dependent on stress-induced secretion of glucocorticoids. *British Journal of Pharmacology*.

[22] Maximiano ES, Elsas PX, De Mendonça Sales SC (2005). Cells isolated from bone-marrow and lungs of allergic BALB/C mice and cultured in the presence of IL-5 are respectively resistant and susceptible to apoptosis induced by dexamethasone. *International Immunopharmacology*.

[23] Iwasaki H, Mizuno S, Mayfield R (2005). Identification of eosinophil lineage-committed progenitors in the murine bone marrow. *Journal of Experimental Medicine*.

[24] Nishinakamura R, Miyajima A, Mee PJ, Tybulewicz VLJ, Murray R (1996). Hematopoiesis in mice lacking the entire granulocyte-macrophage colony-stimulating factor/interleukin-3/interleukin-5 functions. *Blood*.

[25] Mucida D, Kutchukhidze N, Erazo A, Russo M, Lafaille JJ, Curotto De Lafaille MA (2005). Oral tolerance in the absence of naturally occurring Tregs. *Journal of Clinical Investigation*.

[26] Nagatani K, Dohi M, To Y (2006). Splenic dendritic cells induced by oral antigen administration are important for the transfer of oral tolerance in an experimental model of asthma. *Journal of Immunology*.

[27] van den Berg H, Greuter M, Kraal G, den Haan JMM (2010). Different mechanisms regulate CD4^+^ T cell independent induction of oral and nasal tolerance of CD8^+^ T cells. *Immunobiology*.

[28] Arnaboldi PM, Roth-Walter F, Mayer L (2009). Suppression of Th1 and Th17, but not Th2, responses in a CD8^+^ T cell-mediated model of oral tolerance. *Mucosal Immunology*.

